# Learning-based intelligent trajectory planning for auto navigation of magnetic robots

**DOI:** 10.3389/frobt.2023.1281362

**Published:** 2023-12-11

**Authors:** Yuanshi Kou, Xurui Liu, Xiaotian Ma, Yuanzhuo Xiang, Jianfeng Zang

**Affiliations:** ^1^ Laboratory for Soft intelligent Materials and Devices, School of Integrated Circuit, Huazhong University of Science and Technology, Wuhan, China; ^2^ Wuhan National Laboratory for Optoelectronics, School of Integrated Circuit, Huazhong University of Science and Technology, Wuhan, China; ^3^ State Key Laboratory of Intelligent Manufacturing Equipment and Technology, Huazhong University of Science and Technology, Wuhan, China

**Keywords:** precise surgery, small-scale robot, electromagnetic control, learning-based trajectory planning, long short-term memory neural network

## Abstract

**Introduction:** Electromagnetically controlled small-scale robots show great potential in precise diagnosis, targeted delivery, and minimally invasive surgery. The automatic navigation of such robots could reduce human intervention, as well as the risk and difficulty of surgery. However, it is challenging to build a precise kinematics model for automatic robotic control because the controlling process is affected by various delays and complex environments.

**Method:** Here, we propose a learning-based intelligent trajectory planning strategy for automatic navigation of magnetic robots without kinematics modeling. The Long Short-Term Memory (LSTM) neural network is employed to establish a global mapping relationship between the current sequence in the electromagnetic actuation system and the trajectory coordinates.

**Result:** We manually control the robot to move on a curved path 50 times to form the training database to train the LSTM network. The trained LSTM network is validated to output the current sequence for automatically controlling the magnetic robot to move on the same curved path and the tortuous and branched new paths in simulated vascular tracks.

**Discussion:** The proposed trajectory planning strategy is expected to impact the clinical applications of robots.

## 1 Introduction

Untethered robots with the capacity to swim in narrow and winding environments due to their small size and high flexibility have gained great promise in precision medicine, such as drug delivery, minimally invasive surgery, and precise diagnosis. In recent years, many actuation methods for untethered robots have been developed, mainly including optical (Liu et al., 2010; Dai et al., 2016; Palagi et al., 2016; Muiños-Landin et al., 2021), acoustic (Nadal and Lauga, 2014), biological (Magdanz et al., 2014; Hosseinidoust et al., 2016; Zhang et al., 2023), chemical (Solovev et al., 2009; Mei et al., 2011; Sánchez et al., 2015), and magnetic actuation methods (Li et al., 2018; Kim et al., 2019; Wang et al., 2020a; Culha et al., 2020; Yang and Zhang, 2020; Schaff et al., 2022; Li et al., 2023).

For example, microrobots consisting of photoactive liquid-crystal elastomers can be driven by structured monochromatic light (Palagi et al., 2016); small metallic devices were self-propelled by catalytic reactions in fluids for chemical actuation (Mei et al., 2011). Moreover, micro/nanorobots can be integrated with motile organisms to realize biological powered motion.

Magnetic actuation of medical robots has great potential in the diagnosing, precise drug delivery and minimally invasive surgery due to its unprecedented properties including fast response, remote control, and safe manipulation. Therefore, the actuation technology is intensively investigated to achieve effective and precise control. The common magnetic control methods involve permanent magnets (Son et al., 2021), Helmholtz coils (Maxwell coils) (Yu et al., 2010; Zhang et al., 2010; Basar and Ahmad, 2016; Wang et al., 2020b; Jiang et al., 2021), systems consisting multiple electromagnets (Kummer and Abbott, 2010; Niu et al., 2017; Yang et al., 2019; Go et al., 2020), and magnetic resonance (Le et al., 2016; Mutlu et al., 2021; Tiryaki and Sitti, 2021).

In the future, automatic control is hoped to complement or replace the manual operation in the field of minimally invasive surgery, because the manual operation requires long-term training to achieve technique and proficiency, which raises the high threshold and inevitable mistakes. Although fully learning the complex 3D endovascular environments, whether through physical modeling, control algorithms or neural networks, is complex and need a great deal of work, still many automatic control methods have been investigated in 2D environments to lay the groundwork for control in real medical scenarios in the future (Belharet et al., 2012; Qing and Gao, 2016). Present automatic control methods are mainly based on the path-planning algorithm to find a short path or path with less collision between the start and the end (Sabra et al., 2006; Lim et al., 2020), the model-based trajectory planning to design the motion in detail, and the visual feedback system for error calibration in real-time. For example, Meng et al. (2019), found a simple, low-energy path based on a breadth-first search and genetic algorithm and combined sliding mode control, back stepping control, and disturbance compensation to the navigation control system for detailed planning, adding a feedback system for error calibration. In the common detailed and time-related trajectory planning process, it is necessary to model the relationship between the driven current of the electromagnetic actuation system and the motion generated by the magnetic field (Long et al., 2015; Li et al., 2019; Yuan et al., 2019). However, the precise modeling between the magnetic actuation system and the resulting motion is challenging because the magnetically controlled motion always involves many delays including motion inertia (Fruchard et al., 2019; Meng et al., 2019), hardware delay, and software delay. The inertia delay is defined as the motion of the motile robot being affected by the previous moving status (Tabak, 2018; Tabak, 2019). Therefore, it will affect the modeling because the robotic motion is affected by the previous states. The hardware delay is defined as the current switching rate of the current source equipment that can cause the delay of the generated force on the robot during the movement. Furthermore, software delay always exists between the software and the controller, which is the delay in real-time information transmission. Moreover, because the whole process from planning to navigation needs to be wholly completed when the robots need to navigate under the new paths, the present automatic control process lacks extensibility.

**FIGURE 1:**
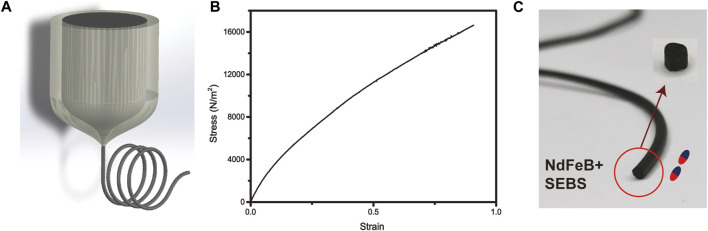
**(A)** The mixture of NdFeB and SEBS is thermally drawn to the elastically magnetic fiber. **(B)** Stress-strain curve of the SEBS mixed with NdFeB. **(C)** The magnetic fiber is magnetized and cut to pieces as the use of untethered robots.

In this paper, we propose a machine learning-based trajectory planning method. By such means, we could use the neural network to directly establish the relationship between the driven current sequence of the electromagnetic actuation system and the trajectory of the robot to generate plans for navigation instead of modeling. In contrast with the conventional kinematics modeling which focuses on each motive state on the path, we aim to establish a global relationship between the current sequence and the consistent coordinates of the whole path. It focuses on the overall process and involves the factors that cause errors such as delays and the environment in the training process. Therefore, the trajectory planning considering the error can directly realize error-free automatic navigation. Since the operation of the magnetic particles is a continuous process, where the state of each moment is related to the previous moments, and the neural network used for training needs to have a long-term memory function, we chose the Long Short-Term Memory (LSTM) neural network (Liu et al., 2019; Yu et al., 2019; Sherstinsky, 2020), it can achieve good continuity and learn the connection between different motion states in the time series (Thuruthel et al., 2019; Truby and Santina, 2020). The detail of LSTM is discussed in the Supplementary Material. The overall structure of our design is shown in Figure 1A with the application scenarios in the cardiovascular environment.

To investigate the above coordinate-current relationship, we realize the learning-based trajectory planning in reference to “learning from demonstration (Tsai et al., 2020)” on an established track. We start from the repeated manual control of the robot to LSTM network training. After training, the trajectory planning is completed by inputting the desired coordinate sequence into the neural network to predict the appropriate driven current sequence. The current sequence is input into the electromagnetic actuation system to realize automatic navigation.

Furthermore, we expand the training database by performing rotating and symmetrical operations on the original track with corresponding coordinate transformation to extend the prediction to other different and more complicated trajectories without new manual demonstration. When there is flow rate, diameter changes, and bifurcations in the environment, we define the current correction factor to adjust motion. The value of the factors is determined by experiment. The proposed trajectory planning method will improve the accuracy and intelligence of robotic navigation and impact minimally invasive surgery.

## 2 Materials and methods

### 2.1 3D printing and robot fabrication

We used the semi-open orbit instead of the restrained soft pipe to provide robots with more space for movement to show controllability. To simulate real and complex vascular structures, we drew the complicated path in “SolidWorks” and printed the track by 3D printing. The printing material we use is polylactic acid (PLA) and the printing machine we use is “RAISE 3D E2”. The “IdeaMaker” is used for slicing.

The magnetic robot is fabricated by thermoplastically pulling a preform which is made by 80 vol% styrene ethylene butylene styrene (SEBS) and 20 vol% Fe_3_O_4_, because SEBS is soft and has great elasticity. Such material is promising in the fabrication of medically invasive robot. The preform is fed into a furnace where materials soften or melt (Figure 1A). The size of the thermally drawn fiber can be controlled by the drawing temperature, the feeding speed of the preform, and the drawing tension (Yan et al., 2020). In order to demonstrate that the material mixed with magnetic particles has low rigidity and good elasticity, which is promising for applications in biomedical fields, we further measured its stress-strain curve. By calculating the slope of the linear region, the elastic modulus of the SEBS blended with ferromagnetic particles obtained by thermal drawing is 12.6 kPa, shown in Figure 1B. The elastic modulus (Young’s modulus) of human muscles, tissues is generally around 1 kpa (Chen et al., 1996). In our future work, we will further coat hydrogel on the surface of the robot to make it more biocompatible.

The minimum diameter of the fiber can be less than 100 μm, so thermally drawn robots have great potential to enter narrow tissues or blood vessels. In this paper, we aim to investigate the auto control of the untethered robot. Therefore, we first magnetize the fiber along the length direction shown in Figure 1C. Secondly, we cut the fiber to pieces of little cylinders with 1 mm long. The diameter is also controlled to 1 mm during the drawing process. The cylinder is latter used to analyze the auto navigation.

### 2.2 Actuation system

We built an electromagnetic system that can control the magnet with 5 degrees of freedom in space. This actuation system contains eight electromagnets, which consist of four coils in the plane and four in the upper dimension. The iron cores within the coils ensure that they can generate a stronger magnetic field and gradient under a relatively low current (10 A corresponds to a gradient of 1 T/m) by focusing the field lines; therefore, locally increasing the field density. This system can configure different operation modes and parameters by the software. The first operation mode is the manual control using the handle and the second is directly entering the current sequence with time intervals to realize auto control. The magnetic field and gradient are generated by the eight current sources through the electromagnetic induction of the coils. To make sure that the state of the coils and their influence on the magnetic particles is the same for every experiment, we need to calibrate it beforehand. First, it is obvious that the current value needs to be reset to zero before the experiment. Secondly, since the change of current is not instantaneous, the inductive effect produced during the change of current will affect the magnetic field, thus affecting the motion. So, we need to make sure that the inductive effect is the same for each experiment, and we calibrate this by setting the current changing rate to the same value for each experiment. The effect of the calibration is evaluated in the Supplementary Figure S2. Before our auto-control work, we model the relation between the current and the magnetic field shown in Supplementary Material. In our work, we utilize the auto control mode to realize automatic navigation by directly inputting the driven current sequence into the electromagnetic system.

### 2.3 Experimental design

#### 2.3.1 Path planning

**FIGURE 2:**
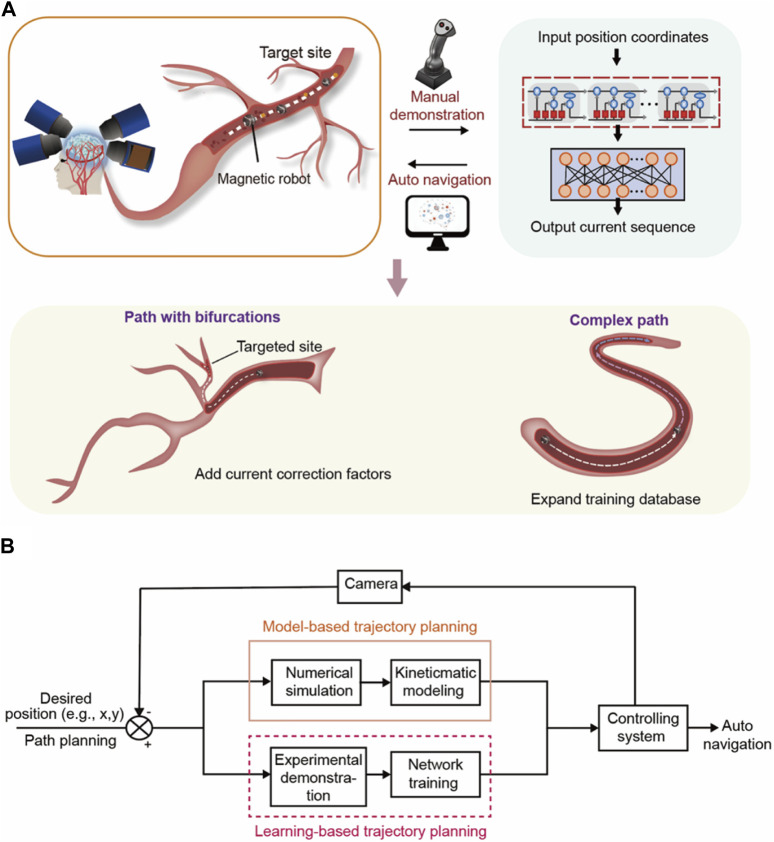
The basic procedure of the learning-based automatic control. **(A)** The overall structure of our work. Automatic control of magnetic microrobots in the cerebrovascular environment based on the magnetic field control system and LSTM neural network. The auto control will start from simple tracks and will be further extended to complex paths and paths with bifurcations. **(B)** The flow chart of our motion planning method compared with the common motion planning process. We proposed a learning-based trajectory planning refer to as “learning from demonstration,” using the manual demonstration and network learning to replace the model-based trajectory planning process.

Figure 2A, the first block shows a hierarchical vascular structure with multiple bifurcations. The complete motion planning process can be divided into two steps, path planning, and trajectory planning, according to the flow chart in Figure 2B. Path planning is the global-scale planning method in the whole motion planning process. It establishes an overall direction for the microrobot. Such overall planning remains blank space between two adjacent track points, which need detailed time-based planning and combined with a controlling system. Therefore, after determining the general path, the specific details in the blank space are determined by trajectory planning. In our work, we use the A* algorithm (Dolgov et al., 2008; Wang et al., 2019) to achieve path planning. The A* algorithm is a widely used basic path planning algorithm that describes each path point with the sum of two functions, the cost of moving from the starting point to that point, and the predicted cost of traveling from that point to the end point. The point that minimizes the sum at each step is the suitable path point found. We extract the boundary information of our track and define the starting and ending points of the path. Input the information above into the A* algorithm; then, generate a path consisting of many path points.

#### 2.3.2 Trajectory planning

**FIGURE 3:**
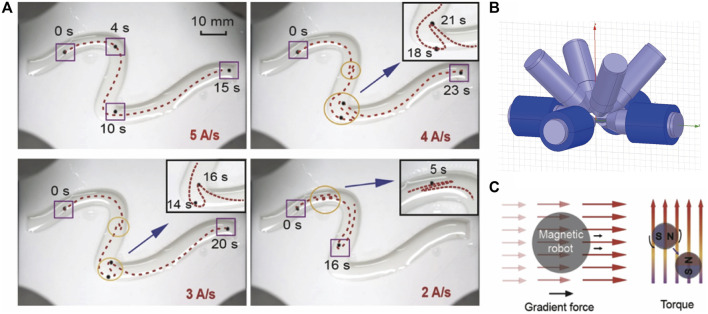
**(A)** Electromagnetically controlled path of microrobot under different current delays. The lower current changing rate corresponds to a larger delay. **(B)** The physical map of the control system consists of 8 coils. We use the 4 coils in the platform to accomplish 2-dimensional control. **(C)** The gradient force and torque exerted on the magnet.

Path planning provides a global direction for path navigation. When considering the movement of robots, to further smoothen the trajectory and avoid collision between robots and the vessel wall, we need the trajectory planning process to design the detailed path in complex vessel sections such as bifurcations and shut turns (Gasparetto et al., 2012; Fruchard et al., 2019; Madridano et al., 2021). Trajectory planning adds a time dimension to the planned path, which is always coordinated with the motion controlling system (e.g., electromagnetic actuation system). For the common navigation method, the controlling process is always accompanied by different kinds of delays we mentioned before and the complicated environment. These delays are not considered in the modeling or controlling process, therefore, the error generated by it needs to be fed back to the input through the feedback system for further error correction. Figure 3A shows the influence of the current source delay. Here, the current delay is defined as a slow current switching rate. The first procedure controls the robot with the lowest current source delay (e.g., 5 A/s). The controlling current sequence is then recorded and applied to different delays. After the current sequence controlling process is completed, the current value remains at the last moment. The trajectories under the larger delays show an obvious mismatch with the original one. When the current switching rate declines to 4 A/s and 3 A/s, the robot experiences irregular loss of direction and deviates from the original trajectory. When the rate further declines to 2 A/s, the original trajectory, and the moving process have completely mismatched because of the high delay, causing the robot not to reach the ending point, as detailed in Supplementary Video S1.

Therefore, according to the red dotted box in Figure 2B, we propose a learning-based trajectory planning method with neural network training that can replace classic model-based trajectory planning to eliminate the controlling error caused by various delays without complicated feedback. In essence, trajectory planning involves inserting detailed intermediate points with time dimensions between the planned path points to navigate the robot along with it. In our work, we complete this process by manually controlling the robot under the desired planned path and use the neural network to learn the current-coordinate relationship from the manual controlling process referring to the idea of “learning from demonstration”. Since the training database based on manual operation includes the delays and environmental factors in the control process, the trained neural network can take the error factors into account and realize low-error trajectory planning.

To control the robot under the actuation system, there are two main types of forces on a magnetic body in the magnetic field: magnetic torque, and magnetic gradient force. When the direction of the magnetic moment of the magnet in the magnetic field is inconsistent with the direction of the magnetic field, the magnetic field will exert a torque on the object so that the magnetic moment is arranged according to the direction of the magnetic induction intensity, as shown in Figure 3C. When the amplitude of the magnetic field is not uniform, the magnet is subjected to a magnetic gradient force in the direction of the increasing gradient. The magnitude of the magnetic gradient force is determined by the magnetic field distribution and the internal magnetic moment of the object, as shown in Eq. 1. We control the robot by the gradient force in this paper.

$$f=\left(m∙\nabla \right)B$$ (1)

The training process of the neural network is shown in Figure 2A, the second block. We first obtain the network training database from manual control by using the handle to navigate the robot in detail between the adjacent path planning points, which is implemented by the manual control mode of the system with 8 electromagnets. Figure 3B shows the physical map of our controlling system. The control current will be recorded by the software and the path coordinates of the robot will be abstracted by MATLAB (Supplementary Figure S4). Because our controlling process is a time sequence in which the state of each time step is affected by the previous time steps, we chose the Long Short-Term Memory Neural Network (LSTM) which can learn long-term dependence to deal with the database by time sequence. Therefore, to eliminate the comprehensive delays we mentioned before and the human uncertainty during the manual demonstration, we next input the prepared training database into the LSTM network. The whole training and prediction process is completed by the Neural Network Training Tool (nntraintool) in MATLAB. Figure 2A, the second block shows the hierarchical structure of the neural network. After the procession of the dropout layer to prevent overfitting. In the LSTM layer, the input sequence is used to train the network. After the LSTM layer, the fully connected layer connects the source eigenspace and the target eigenspace by weighted sum.

In conclusion, the manual demonstration and machine learning (neural network) part are equal to the trajectory planning process which determines the detailed path and controlling method as shown in Figure 2B. The LSTM training replaces the process of kinematics modeling, repeated trajectory planning-feedback-adjustment in the orange square in Figure 2B. The error correction part is integrated with the machine learning process during multiple rounds of training, eliminating the need for real-time feedback for motion calibration. Therefore, the neural network figures out the relationship between the current and motion through repeated training while considering all the inherent errors including the comprehensive delays mentioned before, flow rate, bifurcations, and other complexes. Under such an experimental process, we made the trajectory planning and error correction automatic and synchronous, simultaneously, making the path control continuous and undisturbed, and reduce the image intervention.

## 3 Results

### 3.1 Manual demonstration and forward training

**FIGURE 4:**
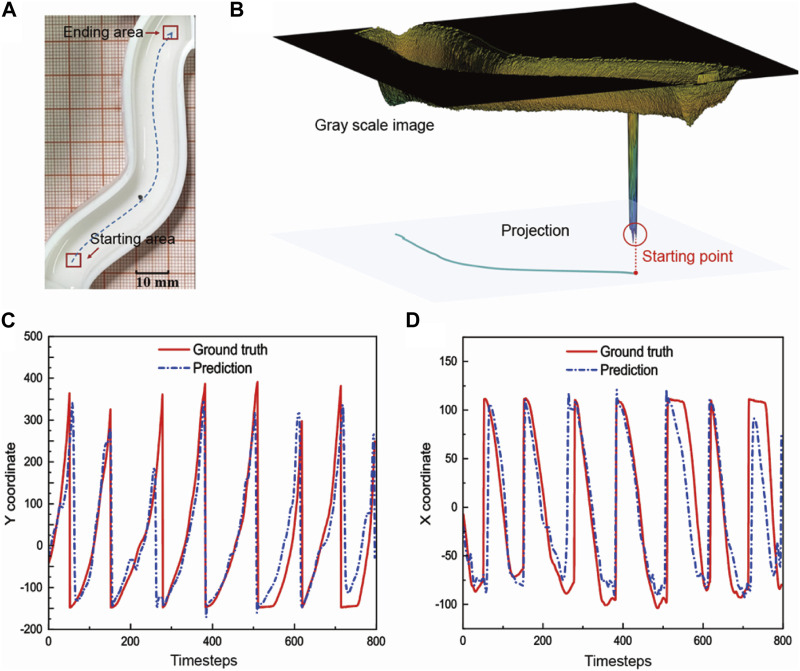
Accuracy evaluation of the network prediction. **(A)** The non-centrosymmetric S-shaped orbit with small curvature. Train the network with the basic path. **(B)** We use the MATLAB code to abstract the moving path of the microrobot. We recognize the microrobot according to the grayscale value difference. **(C)** Predicted value of y coordinates compared with the actual coordinates abstracted by the movement identification code. **(D)** Predicted value of x coordinates compared with the actual coordinates abstracted by the movement identification code.

In the abovementioned trajectory planning method, we aim to use the long short-term memory (LSTM) neural network to predict the current sequence required for automatically controlling the robot stably and accurately. Next, we train the LSTM network with multiple manually controlled processes. Through repeated manual demonstration, the LSTM neural network learns the detailed relationship between current values and position coordinates. To allow the neural network to learn as much as possible about the relationship between robotic movement and current sequence in various positions and directions, and to avoid overmuch training sets, we designed a noncentrosymmetric S-shaped orbit with small curvature, as shown in Figure 4A. The basic training database of the neural network is obtained by manipulating the magnetic robot by the handle in the track.

We find the shortest path based on the path planning algorithm (A* algorithm) according to the starting and ending points and use the handle to manually control the robot along the prescribed path. We turn on the current recording function in the system controlling software, which can export the current values of each current source at 100 ms intervals. The position of the magnetic robot is recorded by the camera, and the position coordinates of the robot are extracted by the motion identification algorithm which is realized by MATLAB codes. Figure 4B shows the path of the manual navigation process abstracted by the difference in greyscale value (Supplementary Figure S5).

We first conform to the order of the manual operations, use the driven current sequence as the input of the neural network, and use the position coordinates of the robot as the output of the neural network to train the network. This step evaluates the accuracy of the prediction of the training network. The detailed procedure and parameters of the LSTM network are shown in the Supplementary Material. After completing the network training, we input the current values in the test database to predict the position coordinates and evaluate whether the trained network can accurately predict the network output. As shown in Figures 4C, D, the comparison between the predicted value of the x and y coordinates and the actual coordinate values recorded by the camera. Multiple cycles represent repeated experimental controls and repeated predictions. We take the time average of the coordinate error and obtain the average error of the x and y coordinates as 3.4% and 4.1%, respectively. The relatively large prediction errors come from the beginning and end of each cycle. There is a sudden change between the end of one round of control and the beginning of the next round. However, such error can be eliminated in real control, because in the actual navigation process when only one set of accurate predictions is needed for a single round of experimental navigation, only one round of predictions is needed. Accordingly, the predicted results match up with the practically recorded coordinates in the experiments, indicating that the trained network can achieve accurate prediction.

### 3.2 Reverse design and analysis

In the abovementioned text, we take the driven current values as network input and position coordinates as output to verify the accuracy of neural network predictions by data analysis. The neural network learns the coordinate changes caused by different current sequences in this way. Next, we need to obtain the current prediction from the LSTM network and evaluate its performance experimentally to verify how the learning-based trajectory planning strategy works. Therefore, in the subsequent training of the neural network, we reverse the input and output, taking the manually controlled position coordinates as the input of the network and the current as of the output. The trained neural network can learn the specific current sequence corresponding to different coordinate changes.

After training, we directly input the desired planned path coordinates of the track to obtain the predicted current sequence. The prediction current values are shown in Supplementary Figure S6. The prediction would be evaluated in the actual experimental navigation. We arrange the current sequence of the prediction result in time series and load it into the control software. The time interval is 200 ms between the adjacent current value and the electromagnet will excite the magnetic field with the driven current, so the robot in the magnetic field will move under the magnetic gradient. We record the moving trajectory of the robot through the camera and further compare it with the manual control path and the standard path planned by the path planning code. The contrasting result indicates that the auto-control is more precise and stable, especially, showing great targeted delivery ability.

**FIGURE 5:**
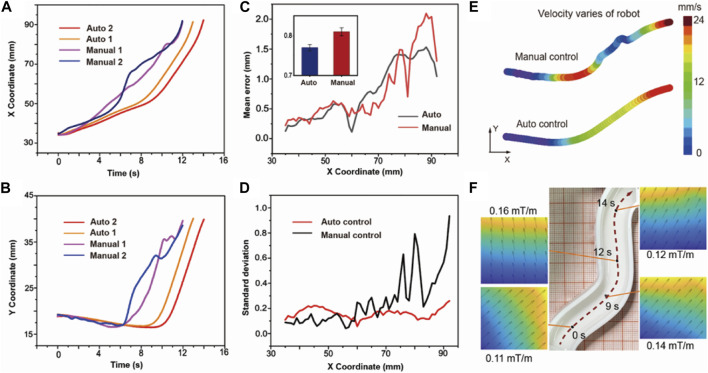
The comparison between the auto and manual-controlled path. **(A)** X coordinates of the microrobot changing with time. The red and orange line represents the auto-control path, and the purple and blue line represent the manual-control path. **(B)** Y coordinates of the microrobot changing with time. **(C)** Mean error between the control path and the standard path. **(D)** Standard deviation of error of the auto-control path and manual-control path. **(E)** Velocity distribution of the microrobot along the path. **(F)** Distribution of the magnetic field in the 10 cm × 10 cm × 10 cm space.

We define the width direction of the track as the X-axis and the length direction as the Y-axis according to Figure 4A. The X-Y position coordinates of auto control, manual control, and the standard line are shown in Supplementary Figure S7. Figures 5A, B show the x, and y coordinates of the path extracted by the MATLAB code varying with time. According to the contrast between the manual and auto path, the changing coordinates of auto-control are smoother with time. Figure 5C evaluates the accuracy of the auto-control and manual-control paths by calculating the spatial deviation between controlled lines and the planned standard line. We define the spatial deviation along the *x*-axis. At each point on the path, the shortest distance between the point and the standard line is defined as the deviation error. Overall, it shows greater accuracy the average error of auto control is 0.76 mm while the manual control is 0.82 mm. The difference between the two error curves reaches its maximum in the ending area of the whole path. The error of the auto-control path at the destination is 1.03 mm in the track width 13 mm, while it is 1.34 mm for the manual-control path, indicating great targeted delivery ability. The specific trajectory is shown in Supplementary Video S2. We further calculate the standard deviation of error. Figure 5D depicts the standard deviation of each control process. The red curve represents the auto-control path, it shows a lower standard deviation compared with the black curve, which indicates that the automatic control has high stability, and each round of the navigation process has tiny fluctuations and gains strong consistency. We also test the spatial distribution of the velocity of the microrobot.

Figure 5E shows the velocity distribution at different path points. We fill the path with different colors to represent different velocities, as marked in the color bar. This shows that under manual control, the color changes irregularly, which means that the robot has unstable acceleration. Under such circumstances, it is difficult for robots to smoothly enter the sharp turn or accurately arrive at the destination. In actual medical surgery, unstable shaking and navigation may also affect the safety and success rate of the surgery. The auto control path, on the contrary, shows a regular change of color. It can be approximately treated as the uniformly accelerated motion, indicating the automatic control generates a relatively average force on the microrobot to ensure a more stable path. In more complex environment, greater stability can ensure that the robot resists more disturbance. The magnitude and direction of the force on the robot can be qualitatively described by the magnetic gradient. To verify the consistency between the force and robotic motion, we analyze the gradient of the magnetic field along the path. We first establish a model of the system consisting of eight electromagnets and simulate the magnetic field. Figure 5F shows the magnetic field along the path at different points marked on the trajectory. The direction of the simulated magnetic gradient is consistent with the moving direction along the trajectory. In the Supplementary Figure S8, we show the field amplitude and gradient in the 10 cm^2^ plane and in a sphere area with a diameter of 5 cm around the robot when it reaches the destination. It depicts the magnetic field distribution in the surrounding space during navigation.

### 3.3 Expansion of the training database

Moreover, to extend our prediction and auto navigation to more complex paths without new and repeated manual demonstration, we perform a series of symmetrical and rotating operations on the initially placed track, see Supplementary Figure S9.

To ensure that the rotating track can completely transplant the spatial magnetic gradient distribution through coordinate transformation, the rotation angle is confined to 90° and multiplied. Each rotating operation allows for further symmetry. When the placement angle of the track changes, we need to transform the manually controlled current data according to the changing position coordinates to expand the position-current data of the training set. Therefore, after enlarging the training database, the training will almost cover the entire 100 cm^2^ plane, ensuring that the neural network can learn the relationship between coordinate changes and driven currents on a larger scale.

**FIGURE 6:**
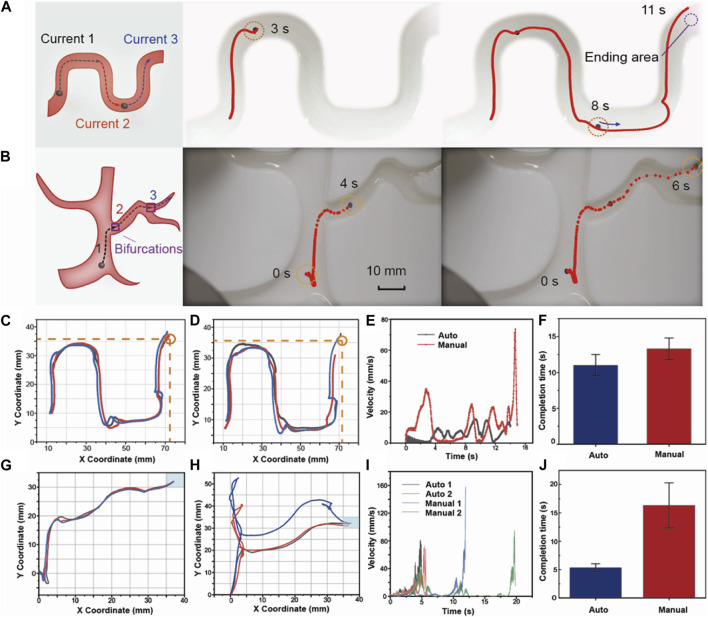
Auto control on complex paths with bifurcations. **(A)** Current prediction and auto navigation on an S-shaped new path without any other manual demonstrations. **(B)** Current prediction and auto navigation on the path with diameter changes and bifurcations. **(C)** Auto control path in the S-shaped track. **(D)** Manual control path in the S-shaped track. We mark the destination coordinate as (72 mm, 36 mm), and draw a circle area with a diameter of 5 mm. **(E)** The arrival rate to the destination of auto-control and manual-control paths. **(F)** Different completion times to reach the destination. **(G)** Auto control path in the track with changes of vessel diameter and bifurcations. **(H)** Manual control path in the track with changes of vessel diameter and bifurcations. **(I)** The velocity changes by time of auto and manual control. **(J)** Different completion times to reach the destination.

After completing more comprehensive training and learning of motion control, we can predict the current sequence under a more complex new path without new manual demonstration. In Figure 6A, we design an S-shaped tortuous track to evaluate the capacity of the LSTM network to extend the prediction to a new, complicated track. We decompose the long complex path into discrete path segments and predict the corresponding current sequence separately to improve the accuracy of each part of the predictions. We divide the “S” path into three segments shown in Figure 6A and integrate the predicted currents of each section to obtain a whole current sequence of the entire path. In Figure 6A, the red dashed line represents the auto-control path, and the robot gradually completes the entire path along the preset segmented paths. The entire motive process is shown in Supplementary Video S3. We also control the robot manually to compare it with the automatically navigated results. Figures 6C, D show the selected auto-control paths and the manual-control paths. The navigating accuracy is evaluated by the destination reaching rate while reaching the circle area is defined as an accomplishment. We control the robot automatically and manually ten times each. The results of the auto navigation show a great destination reaching rate of 80%, while the manual-control result is only 66.6%. In Figure 6D, some selected manual-control paths cannot reach the destination and stop halfway. This is because the magnetic field in the corners of the plane is relatively small and the magnetic gradient exhibits greater inhomogeneity. When the robot goes close to the vessel wall, the adhesive force of the wall on the robot will hinder the movement. Figure 6E selects the recorded paths of auto-navigation and manual control and presents the variation of robot speed with time. In manual control, there will be zero-speed pauses due to the delay of current value switching at the turning. The driven current obtained by neural network trajectory planning makes the robot’s movement consistent and fluent. In Figure 6F, we compare the completion time of auto and manual control processes. The average completion time and fluctuation show that auto navigation under the predicted current gains high efficiency.

### 3.4 Prediction over paths with bifurcations and diameter change

When considering the influences of the environment, it is necessary to consider not only the tortuosity and complexity of the path, but also the influence such as path diameter, bifurcations, wall effect (Arcese et al., 2011; Peng et al., 2017), and flow rate. Under multiple factors, relying only on current predictions of the trained LSTM network may not ensure that the controlling system accurately controls the robot. In Figure 6B, we design a brunched path with changing widths to simulate the diameter changes of the path to further improve the capacity of our network prediction and auto navigation in a more complex environment.

We first discuss the influence of the diameter change of the path. We attribute the bifurcation to the situation of diameter change between the main path and the branches. When the diameter changes, the drag force increases according to the drag coefficient of variation. To solve the influence of the varying drag force on the automatic navigation at different path diameters, we adapt the predicted current to the change in the environment by increasing or decreasing the current value according to the change of the path diameter. The amplified current values will generate an amplified magnetic gradient to overcome the resistance force. We define the current correction coefficient, which is determined by the drag coefficient of variation in paths of different diameters in Eq. 2 (Arcese et al., 2011).

$$\tilde{F_{d}}={\left[\frac{1-\lambda^{\alpha_{0}}}{1+{\left(\frac{\lambda }{\lambda_{0}}\right)}^{\alpha_{0}}}\right]}^{2}∙F_{d}$$ (2)

Parameters *α*_*0*_ and *λ*_*0*_ are functions of the Reynolds number, regularly, they are assigned as constants ( $\alpha_{0}$ = 1.5 $\beta_{0}$ = 0.29). $\tilde{F_{d}}$ denotes the drag force considering the wall effect, the function of *λ* denotes the drag coefficient of variation and *λ* is the function of the radius of the path (r) as shown in Eq. 3.

$$\lambda =\frac{2r}{D}$$ (3)

D denotes the diameter of the path. Different diameter changing ratios will correspond to different values of the current correction coefficient. In the previous article, we mentioned that we can divide the path into multiple sections and predict the driven currents over them. On this basis, we could modify the predicted current of each section of the path by multiplying the current correction coefficient according to the specific path diameter. We experimentally determine the value of the current correction coefficient by designing a series of small tracks to simulate the process of diameter changing from large to small, as detailed in the Supplementary Figure S9. The diameter starts from 15 mm and gradually shrinks. Correspondingly, we use the current that drives the robot to move in the 15 mm diameter as the reference current. We control the volume of the robot to be constant at 1 mm^3^. As the diameter decreases, the control current gradually increases until the robot can enter the narrow path. By recording the relationship between diameter and current value, we can get a series of scattered plots of current versus diameter. To obtain the basic form of the fitting function, we refer to the theory of drag coefficient of variation at different diameters shown in Eq. 2, using the true data of magnetic particles (diameter 1.26 mm), calculating the theoretical coefficient curve, and multiply it by the reference current to form the theoretical current value curve. In Supplementary Figure S9, the comparison of the theoretical results with actual experimental measurements shows that the trends of the current value are generally consistent, and the coefficient curves can also be fitted well based on the theoretical function of the coefficient in Eq. 2.

In the path diagram of Figure 6B, we segment the path before each bifurcation. The whole path is separated into three parts for the LSTM network to generate predictions. According to the numerical relationship between the current correction coefficient and the diameters, the second and third paths require a twofold and fourfold magnification of the predicted current respectively. In the selected auto-control path shown as the red dashed line, the robot reaches the second bifurcation point at 4 s and finally reaches the ending point at 6 s. Intuitively, the robot under automatic navigation has reached the destination according to the path very accurately. To further verify the superiority of automatic navigation, in Figures 6G, H, we compare the completion of the automatic-navigation path and the manual-control path. During manual control, we found that for the path with bifurcations and smaller diameter, it is difficult for a human to control the robot to enter the narrow path accurately and timely at the bifurcation in the face of the delay of control current and human response delay. As shown in Figure 6H, at the bifurcation “two,” the manual path will deviate from the preset path because of the delay of the current change and reaction time of the human. In practical medical application scenarios, it requires technicians to undergo long-term training to achieve precise operation. However, under the automatic navigation in Figure 6G, the navigated path of the robot is very accurate, there is no obvious fluctuation and deviation, and each time the controlling process shows strong consistency. The dynamic and full controlling processes are recorded in Supplementary Video S4. Figure 6I analyzes the distribution of the velocity of each controlling mode. The maximum velocity of the automatic control is approximately 82 mm/s and the velocity changes with time in a continuous upward tendency. On the other hand, the maximum velocity of manual control reaches 160 mm/s, and the velocity fluctuates dynamically during its movement, often switching between high and low speeds. Figure 6J further compares the difference in completion time, showing that the auto navigation method could save 65.6% of the time compared with manual control which can greatly improve the controlling efficiency.

### 3.5 Influence of the flow rate on robotic navigation

Modeling the fluidic-flow environments is a very complex task (Arcese et al., 2011; Peng et al., 2017; Li et al., 2023). Since the flow velocity of the fluid in the limited vessel diameter changes parabolically along the vessel diameter, the parabolic distribution of the flow velocity will also be distorted and deformed with the change in space when turning or passing through the bifurcation, and finally be restored in the stable region as a parabola. Therefore, when analyzing automatic control, complex algorithms are usually used to analyze the influence of flow velocity on motion, such as the model-free disturbance observer-based controller (Sabra et al., 2006), which is specially designed for environmental uncertainty.

In this paper, we treat the parabolic-distributed velocity of the fluid as a whole for simplified analysis and use the velocity at the center of the fluid to represent the integral flow rate. We define the “additional current” to counteract the effect of the flow rate by applying a certain current value. In the navigation with the flow rate, we add additional current to the originally predicted current, to counteract the influence of the current, and make the robot move according to the planned trajectory. We experimentally measure the specific values of the additional current at different given flow rate values and adjust the current until the robot can remain relatively stationary with the track in the flow. We performed multiple sets of experiments to measure the value of the additional current, and the relationship between different flow rates and the additional current is shown in the Supplementary Material, as shown in Supplementary Figure S9.

In conclusion, we test the current correction factors and additional currents of many diameters and different values of flow rate to obtain an accurate fitting curve. We chose points away from the existing experimental points and reached the maximum current of the current source, as shown in Supplementary Figure S9 for verification. We see that the test points match up with the fitting lines, indicating the good universality of the result.

In the future, we plan to further solve the influence of the blood by replacing the fluid used for training in this article with real blood and replacing the 3D-printed path with a specially designed blood vessel model that is more biologically compatible. In practical medical applications, the LSTM can be pre-trained in classical vascular environments such as simulated carotid arteries, thigh arteries, and cerebral blood vessels using the LSTM. A large amount of pre-training can form a huge database, and when it is necessary to control in a new environment, real-time information can be added combined with the historical data, so that in the face of new vascular paths or the same vascular paths of different people (e.g., two people need the medical robots to travel the path of the carotid artery, but due to individual differences, the blood flow rate, hematocrit rate are different), there can be an available database that can be used as a reference at any time.

## 4 Discussion

In this paper, we propose a learning-based trajectory planning method to realize the automatic navigation of magnetic robots and solve the influence of various uncertain delays such as inertia delay, hardware (current source) delay, and software delay. We realize trajectory planning by training the neural network with manual operations. The trained network is validated by numerical testing and experimental testing. The first takes the current sequence as the network input and the coordinates as the output, obtaining the average predicting error of the coordinate of x and y for 3.4% and 4.1%, respectively. The second sets the position coordinates as network input and the current values as output. Since the current sequences in the training database are not the sequences that can perfectly control the robot, it is necessary to use the predicted output current of the network to control the robot experimentally, and the accuracy of the prediction and the feasibility of our trajectory planning method are judged by the controlling results. For the comparison between auto control and manual control, the path of automatic navigation is closer to the standard path with an average error of only 0.76 mm while the manual navigation is 0.82 mm, the standard deviation is smaller, and the speed distribution is relatively stable, which can be approximated as uniform acceleration along the trajectory.

Further study extends our predictions to other more complex tracks without other new manual demonstrations by performing rotation and symmetry operations on the originally placed track to expand the training database. Moreover, when there are bifurcations, diameter changes, and flow rates in the tracks, we define the “current correction coefficient” and “additional current” to optimize the predicted current sequence to adapt to more complex environments. The effect is verified by auto navigation on the tortuous S-shaped path and the branched path. In the ten automatic controls on the s-shaped path, the robot’s arrival rate toward the ending point reached 80%. For comparison, we also manually control the robot with an arrival rate of 66.7%. For the navigation in the branched path, the velocity of the automatic control is relatively regular in a continuous upward tendency and could save 65.6% of the time compared with the manual control to reach the destination which can greatly improve the controlling efficiency. Finally, the “additional current” is measured to keep the robot relatively static in the flow-through experiment. In actual control, the additional value of current can be added to the original prediction current to offset the effect of the flow rate.

Overall, the proposed learning-based trajectory planning method can eliminate the effects of delays and can be applied to predict arbitrary complex paths. In further investigation, we can apply more environmental influences in the training procedure to realize more complex robotic navigation in more complicated environments.

## Data Availability

The raw data of this study are available on https://github.com/ysKou/LSTM_database.
