# Supplementary material for: Learning-based intelligent trajectory planning for auto navigation of magnetic robots
Source: Front Robot AI. 2023 Dec 11;10:1281362. doi: 10.3389/frobt.2023.1281362 (PMC10750377; doi:10.3389/frobt.2023.1281362)
Supplement: Supplementary file 2 [file DataSheet1.pdf]

Supplementary Material

Supporting Videos description

Supporting_Video1.mp4: Navigation of the magnetic robot under different current switching rates.

Supporting_Video2.mp4: Navigation of the magnetic robot on the original simple path.

Supporting_Video3.mp4: Navigation of the magnetic robot on the S-shaped path.

Supporting_Video4.mp4: Navigation of the magnetic robot on the complex path with bifurcations and diameter changes.

1 Supplementary Data

1.1 Parameters of the neural network

Before training, we perform the standard and non-dimensional process of the input data set. As shown in Eq. 1. x_i refers to every sample, $\mu(x)$ refers to the mean value of this matrix, and $\sigma(x)$ refers to the standard deviation of the matrix.

$$Z(x_i) = \frac{x_i - \mu(x)}{\sigma(x)} \tag{1}$$

Neural network parameters: Input features: 2, Output features: 4, Hidden units: 100 , Dropout rate: 0.05, Optimizer: Adam, Max epochs: 2000, Initial learning rate: 0.005.

After each 125 epoch, the learning rate is multiplied by a 0.2 factor.

2 Supplementary Table

	Network type	Inter-neural effect	Memory function
**CNN**	feedforward	no	no
**RNN**	feedback	through hidden state “h”	shot-term memory
**LSTM**	feedback	through hidden state “h” and cell state “c”	long-term memory

Table 1

The comparison among CNN, RNN and LSTM.

The common neural network includes the Convolutional Neural Network (CNN), Recurrent Neural Network (RNN), and LSTM. CNN is a kind of Feedforward Neural Network, whose input of time “t” is not relevant to the input of time “t+1”. It is often used to solve image recognition because there is no time relevance within. CNN and LSTM both have a memory function, which means neurons can receive not only signals from other neurons, but also their own feedback signals. They belong to the Feedback Neural Network, however, due to the gradient dispersion, it is not possible for RNN to update the learning parameters of the earlier time step according to the gradient in the later time step. Therefore, RNN does not have long-term memory, but only short-term memory. The LSTM contains a new structure called the cell state explained as the “memory” of the former data. In contrast with the RNN which only has one activation function (tanh, sigmoid), LSTM proposes new gate mechanisms, including forget gate, input gate, and output gate. The forget gate and the input gate filter the hidden state and the input information through the sigmoid function respectively, screening the valid information to be memorized and forget the useless information. The updated cell state is equal to the sum of the information from the cell state at the previous time filtered through the forget gate and then superimposed on the input gate. Therefore, the structure of LSTM effectively solves the short-term restriction of RNN and has long-term memory.

3 Supplementary Figures

3.1 LSTM neural network

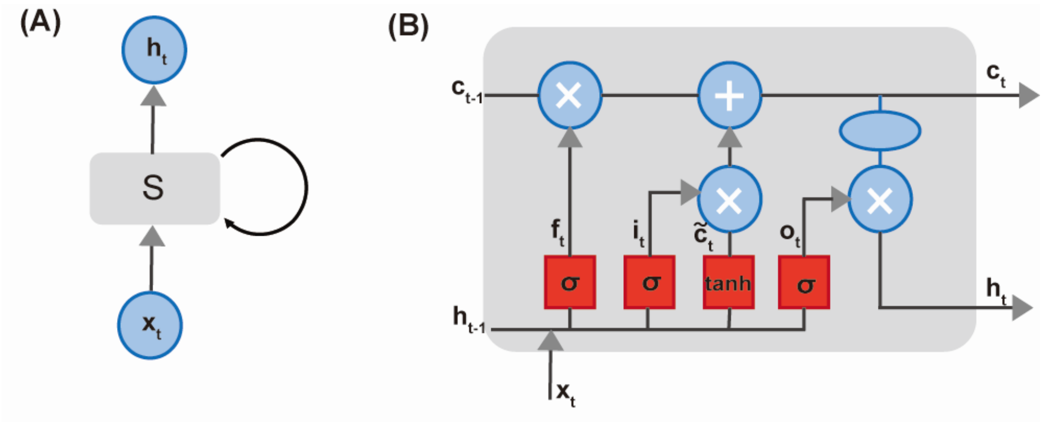

Supplementary Figure 1

(A) RNN network. **(B)** LSTM network.

The structure of the LSTM network is more complex than RNN, it introduces the cell state and use the forget gate, input gate and output gate to control the data. As shown in the figure S, the forget gate filters the input of time "t" and the output of time "t-1" by the following function (W refers to the weights, b refers to the bias).

$$f_t = \sigma(W_f \cdot [h_{t-1}, x_t] + b_f) \quad (2)$$

If the data becomes 0 after the sigmoid function, it is also 0 in subsequent multiplicative relational operations and is therefore forgotten. If its value is 1, it is considered as important information and remains memorized. The input gate primarily controls the selective addition of x_t to the cell state as shown in the following function.

$$i_t = \sigma(W_i \cdot [h_{t-1}, x_t] + b_i) \quad (3)$$

$$\tilde{c_t} = tanh\ (W_c[h_{t-1}, x_t] + b_c) \quad (4)$$

The sigmoid function selectively discards feature dimensions with an output of 0. *tanh* is equivalent to the activation function in RNN and performs a linear transformation for the original input. The resulting $\tilde{c_t}$ and f_t together update the current cell state. Similarly, the output gate also requires selective memorization

and forgetting of the output information.

$$o_t = \sigma(W_0 \cdot [h_{t-1}, x_t] + b_0) \qquad (5)$$

Although LSTM is more complex compared to RNN and needs to learn a lot of parameters, it has a long-term memory function thus can effectively train long-term time-correlated sequences.

3.2 Modeling and simulation of the coils with cores

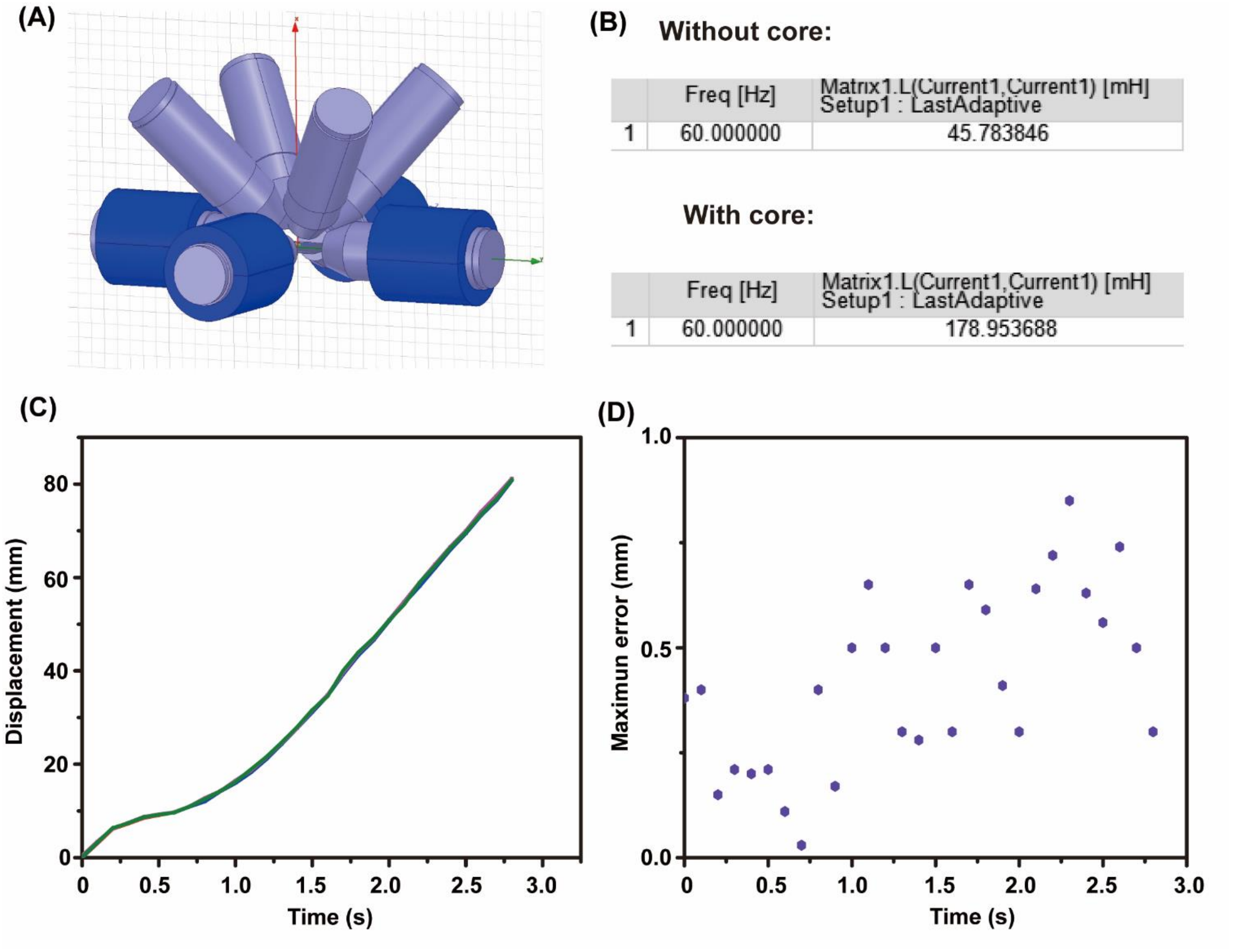

Supplementary Figure 2

(A) Modeling of the coils in the ANSYS. **(B)** The inductance of the coil with and without the core. **(C)** Repetition of the control five times at the same current, current changing rate, and orbit, with a 5 min interval between each control. (D) The maximum error of position at different time among the 5 times' control.

According to the theoretical calculations, according to the supplementary eq.6, we get the ideal value of

$$L = \mu_0 N^2 A/l \tag{6}$$

inductance of the coil without the core. *N* refers to the number of turns (960), *A* refers to the cross-sectional area (*r*=42.75 mm) and l refers to the length of solenoid (140 mm). The caculating value is 47.49 mH. Compared to the simulated value, the error rate is 3.74%, therefore, we can estimate the inductance of the coil with core to be 178.95 ± 6.68 mH.

To make sure that the state of the coils and their influence on the magnetic particles is the same for every experiment, we need to calibrate it beforehand. First, it is obvious that the current value needs to be reset to zero before the experiment. Secondly, since the change of current is not instantaneous, the inductive effect produced during the change of current will affect the magnetic field, thus affecting the motion. So, we need to make sure that the inductive effect is the same for each experiment, and we calibrate this by setting the current changing rate to the same value for each experiment. To verify the validity of this calibration, we repeat the control of the same magnetic particles in the same orbit, applying the same current, setting exactly the same current changing rate, and obtaining displacement-time curves. As can be seen from Sup. Fig. C, the motion of the magnetic particles is almost the same in 5 times of control, and the error in Fig. D also shows that the maximum position deviation is only 0.74 mm in 5 times of control, which indicates that this calibration can make the state of the instrument remain basically the same in each experiment.

3.3 Modeling of the control system

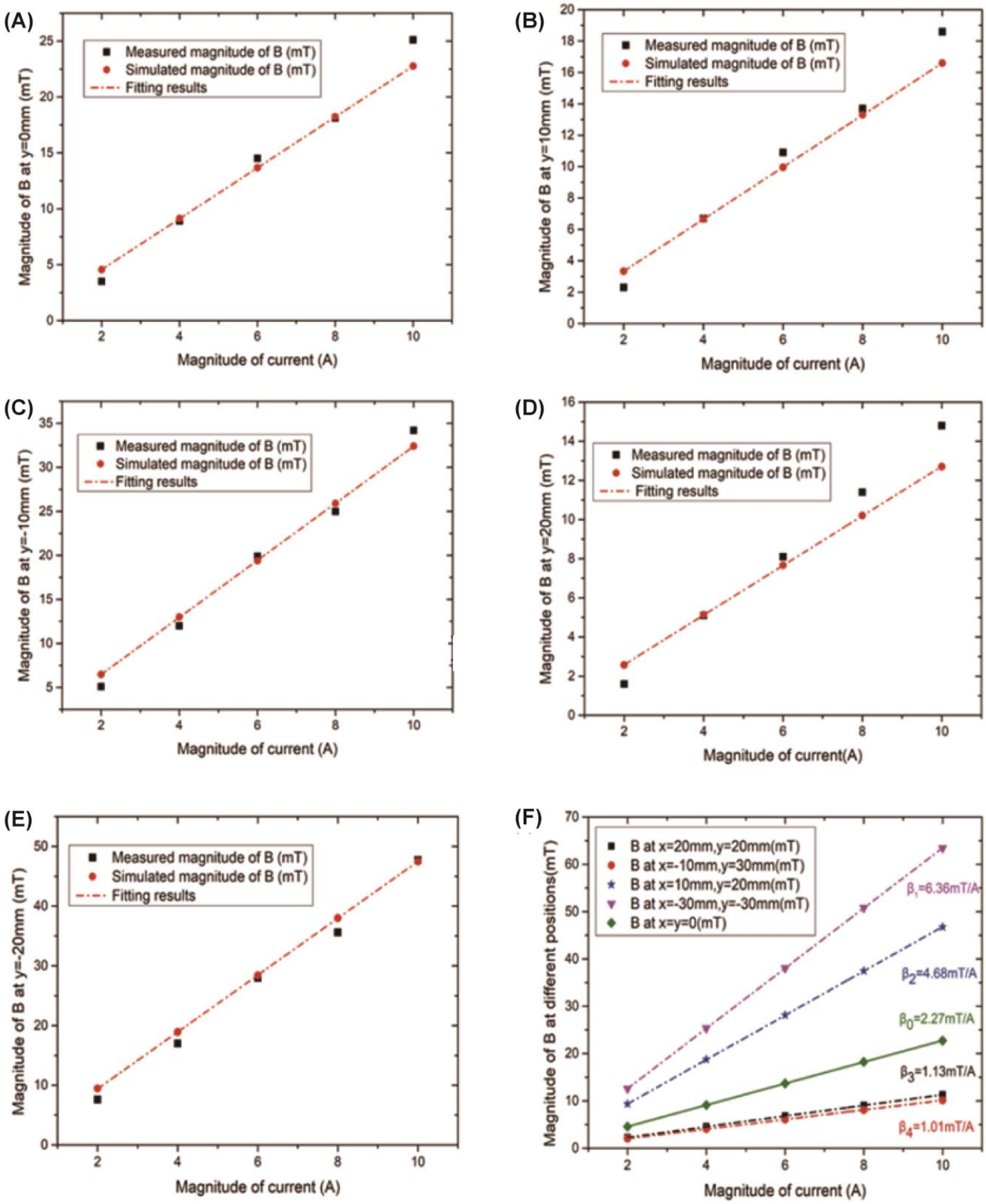

Supplementary Figure 3

(A)-(E) The comparison among the measured, simulated magnitude and the fitting result at different points.

(F) The ratio coefficient between B and current at different points.

We use theoretical modeling to obtain the relationship between the in-plane magnetic field and the input current of the actuation system.

To establish the mapping relationship between the in-plane magnetic field and the input current, we define the coefficient matrix I_K. When there is only one electromagnetic coil working, set its current as I_1, the magnetic field generated in the plane is defined as the magnetic field matrix B_1, and the magnetic field intensity at any point in the plane is B_{i1} (which is an element in the matrix). The corresponding relationship was $B_{i1}=I_{Ki}I_1$ (where I_{Ki} represents an element in the coefficient matrix I_K). Therefore, every points in the plane have specific B-I coefficients defined as "$B_{i1}=I_{Ki}I_1$", the relationship between the current and the magnetic amplitude in the plane can be written by a matrix as "$\boldsymbol{B_1}=\boldsymbol{I_K I_1}$". Divide the 100 cm^2 plane every 1mm, and the obtained magnetic field matrix is a 101×101 matrix. Because the data we use for theoretical modeling are all from the software simulation results, we need to compare the simulation magnetic field with the experimental measurement results to prove the data rationality of theoretical modeling. After input a certain current to the current source, we used Gauss meter and simulation software (ANSYS) to obtain the amplitude and direction of the magnetic field at each point in the plane respectively.

After the coefficient matrix $\boldsymbol{I_{K1}}$ corresponding to I_1 is obtained, we can obtain the other three "magnetic field-input current" relationships and the coefficient matrix when the other three current sources work independently and respectively. Finally, through simple superposition calculation, the magnitude of the magnetic field at any point when the four electromagnetic coils in the plane work at the same time can be obtained. Finally, we superimpose the magnetic fields generated by the four current sources to obtain the B_x and B_y components of the magnetic fields generated at each point in the plane when the four current sources work simultaneously. Then, the magnitude and direction of the magnetic field can be obtained through vector synthesis.

We also need to reserve the above modeling process. When the magnetic field of one point is known, we need to calculate the corresponding current value of each current source.

The following two sets of equations describe the relationship between the magnetic field strength generated by each coil at different positions and the current I. To solve it inversely, we have already known the position and the sum of the components of B_x and B_y respectively. Therefore, we need to solve a set of suitable current combinations. We use MATLAB to solve the indefinite Equation as follows.

$$
\begin{aligned}
B_{X1} &= I_1 I_k \cos\varphi(x,y)\cos\theta(x,y) \\
B_{X2} &= -I_2 I_k \cos\varphi(y,-x)\, sin\theta(y,-x) \\
B_{X3} &= -I_3 I_k \cos\varphi(-x,-y)\cos\theta(-x,-y) \\
B_{X4} &= I_4 I_k \cos\varphi(-y,x)\, sin\theta(-y,x)
\end{aligned}
\tag{7}
$$

$$
\begin{aligned}
B_{y1} &= I_1 I_k \cos\varphi(x,y)\sin\theta(x,y) \\
B_{y2} &= I_2 I_k \cos\varphi(y,-x)\, cos\theta(y,-x) \\
B_{y3} &= -I_3 I_k \cos\varphi(-x,-y)\sin\theta(-x,-y) \\
B_{y4} &= -I_4 I_k \cos\varphi(-y,x)\, cos\theta(-y,x)
\end{aligned}
\tag{8}
$$

3.4 The recorded currents of the manual control in the original track

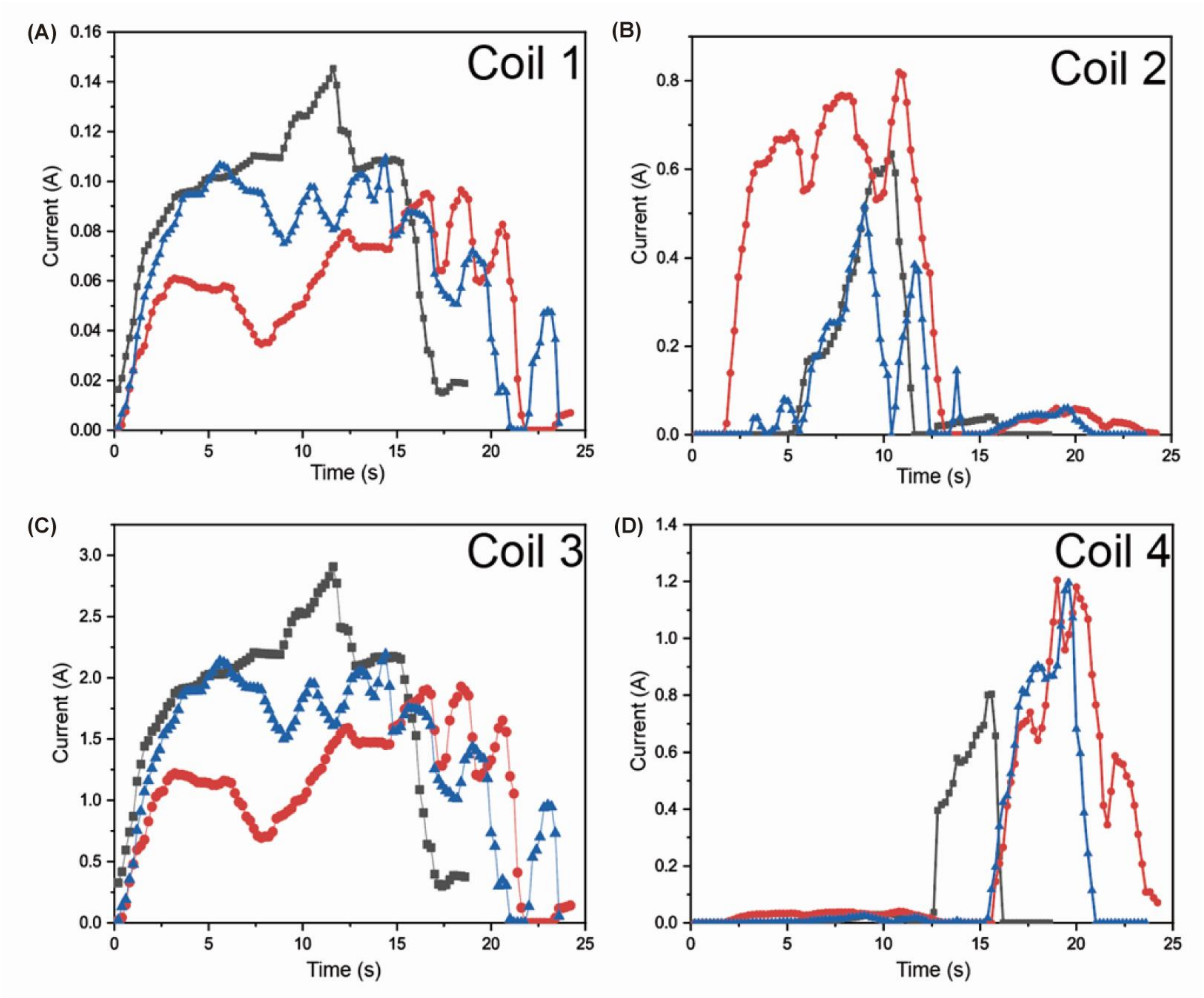

Supplementary Figure 4

The recorded currents of 4 coils under manual control. **(A)** the recorded currents of the first coil. **(B)** the recorded currents of the second coil. **(C)** the recorded currents of the third coil. d) the recorded currents of the fourth coil.

We selected three sets of manually controlled processes and use the current recording function of the controlling software to record the manual-control currents.

3.5 Motion recognition

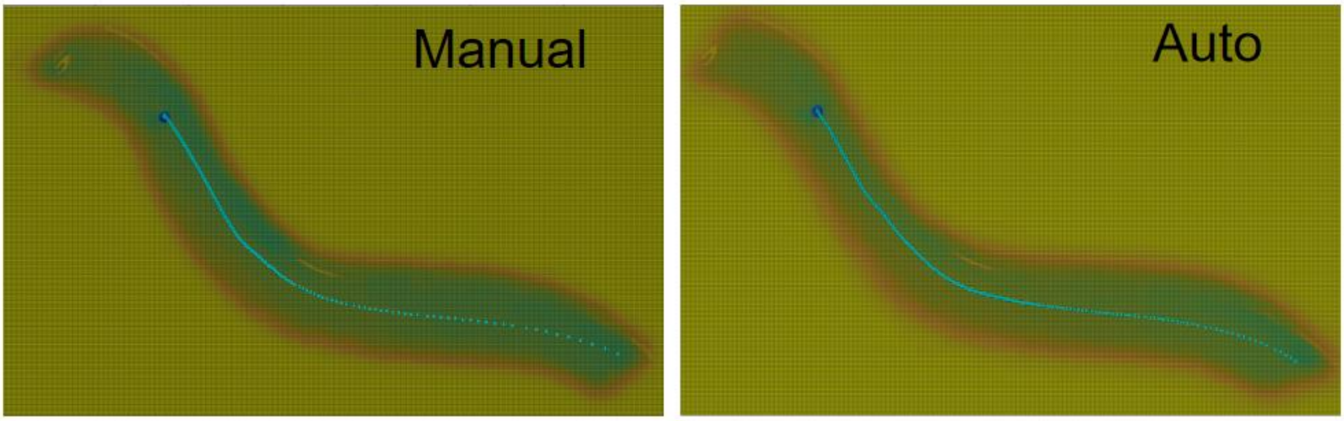

Supplementary Figure 5.

The trajectory maps of auto and manual control results reconstructed by the MATLAB code.

We identify robots by the difference in gray values. When the robot moves under the control of the electromagnetic actuation system, the camera is used to catch the movement process. We use MATLAB code for video processing. First, we convert the colored image of every frame of the video into a grayscale image. Second, we find the robot in the grayscale image according to the gray value difference and mark the centroid of the robot. Finally, we record the position coordinates through the MATLAB code, and calculate the speed according to the previous and the next frames. If there are noises around the recognized object, we further calculate the area of the object and the noise and delete the area whose area is less than a certain value, and finally leave only the object that we want to be recognized. Finally, the position of each frame is superimposed to form a complete path.

3.6 Prediction of the current sequence

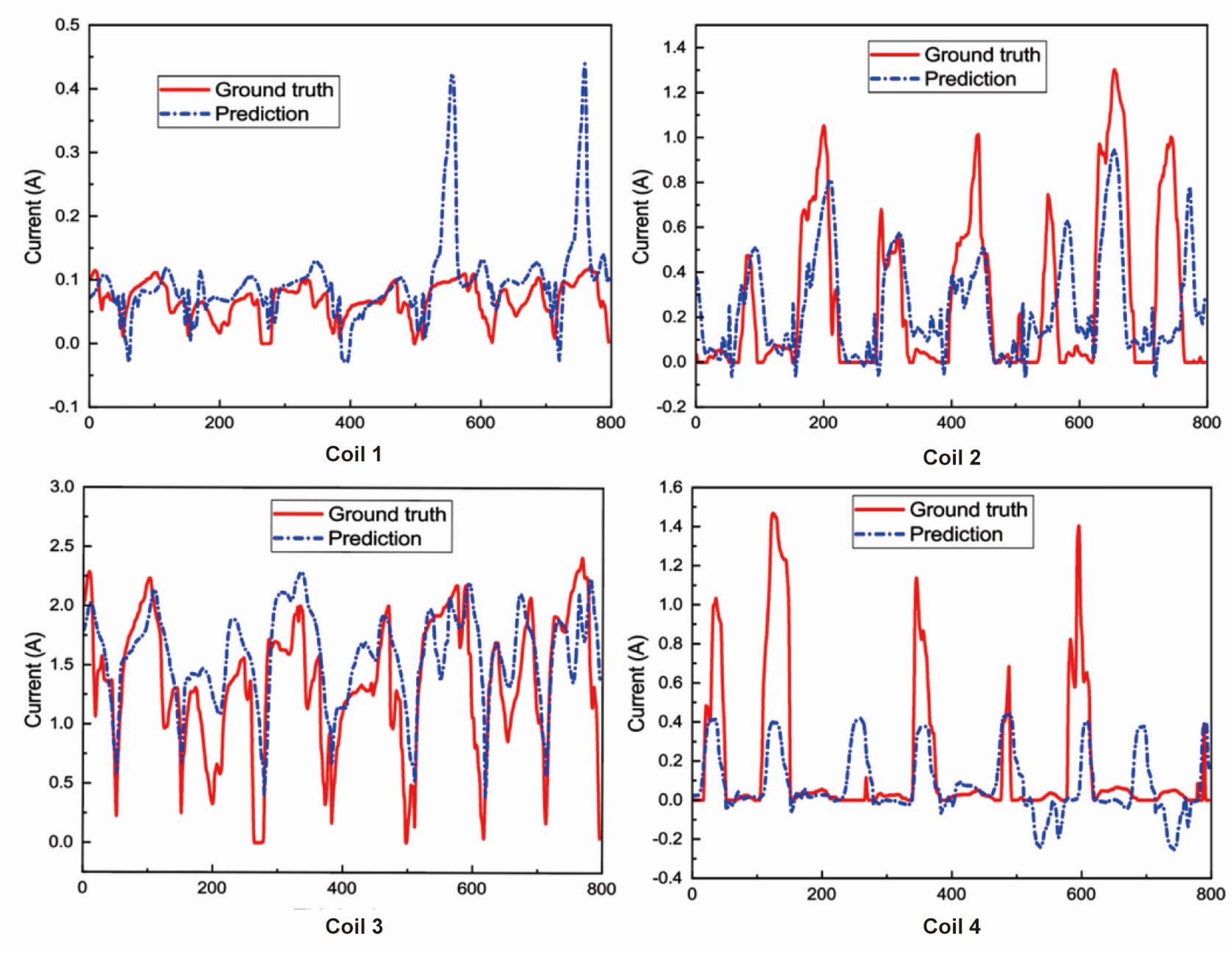

Supplementary Figure 6

The prediction result of the 4 coils.

Supplementary Figure 4 shows the prediction results of the current sequence of the controlling system. We analyze the difference between the predicted current value and the current abstracted from manual operation. As shown in Figure 4., coil 1, coil 2, coil 3, and coil 4, are the predicted current values of four electromagnet coils respectively. The positions of the peaks are relatively the same, however, the amplitudes are not similar to the manual controlling current sequences. This is because the manual control current controlled by the handle is not accurate due to various errors such as the delay and the human uncertainty. It can be seen that the peak of the predicted current is always lower than the manual control current. It is an

ideal phenomenon because the robot doesn’t need much current to drive, such a lower current may ensure the robot moves more smoothly. However, the practical evaluation of the predicted current will be implemented in experiments, by adopting the current sequences for automatic control.

3.7 The trajectory on the original path

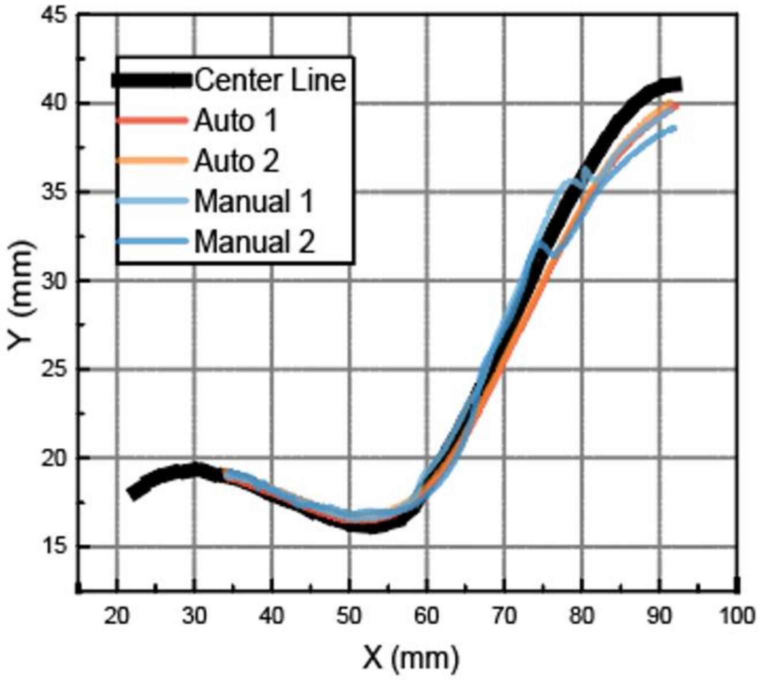

Supplementary Figure 7

The trajectories of the auto navigation path, manual navigation path and the center planned path.

3.8 Distribution of magnetic field and gradient

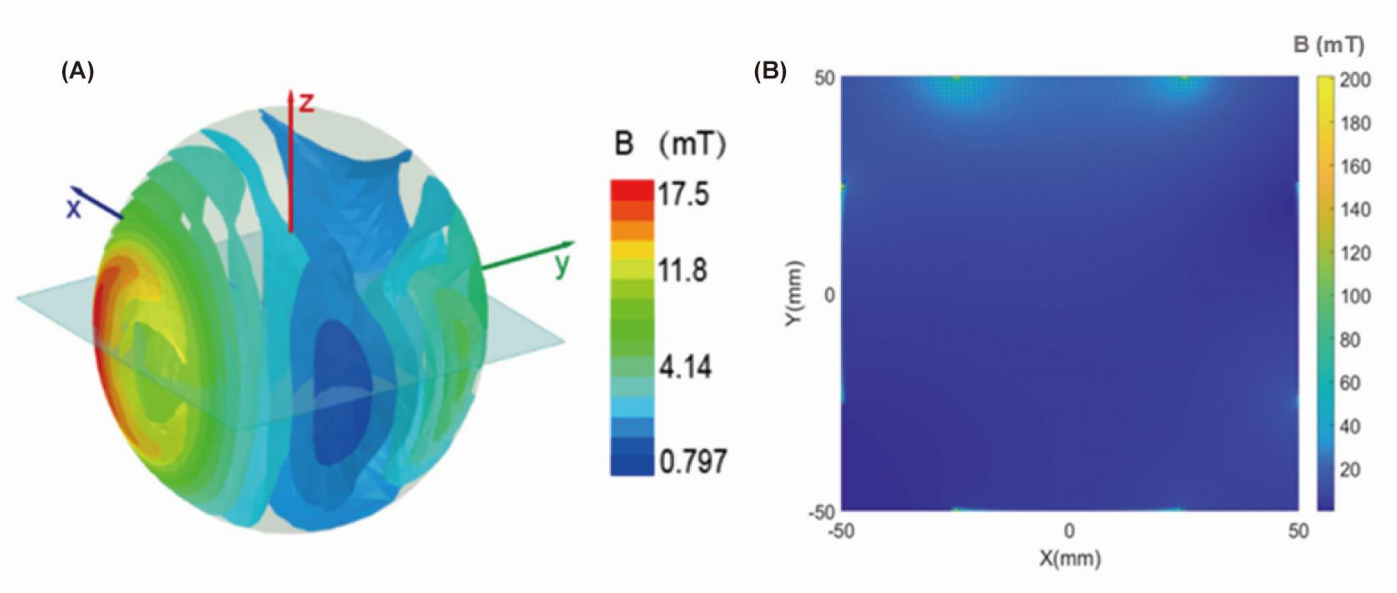

Supplementary Figure 8

(A) The distribution of magnetic field in the sphere of radius 5 cm when the robot approaches the ending point. **(B)** The amplitude of the magnetic field in the 100 cm^2 plane.

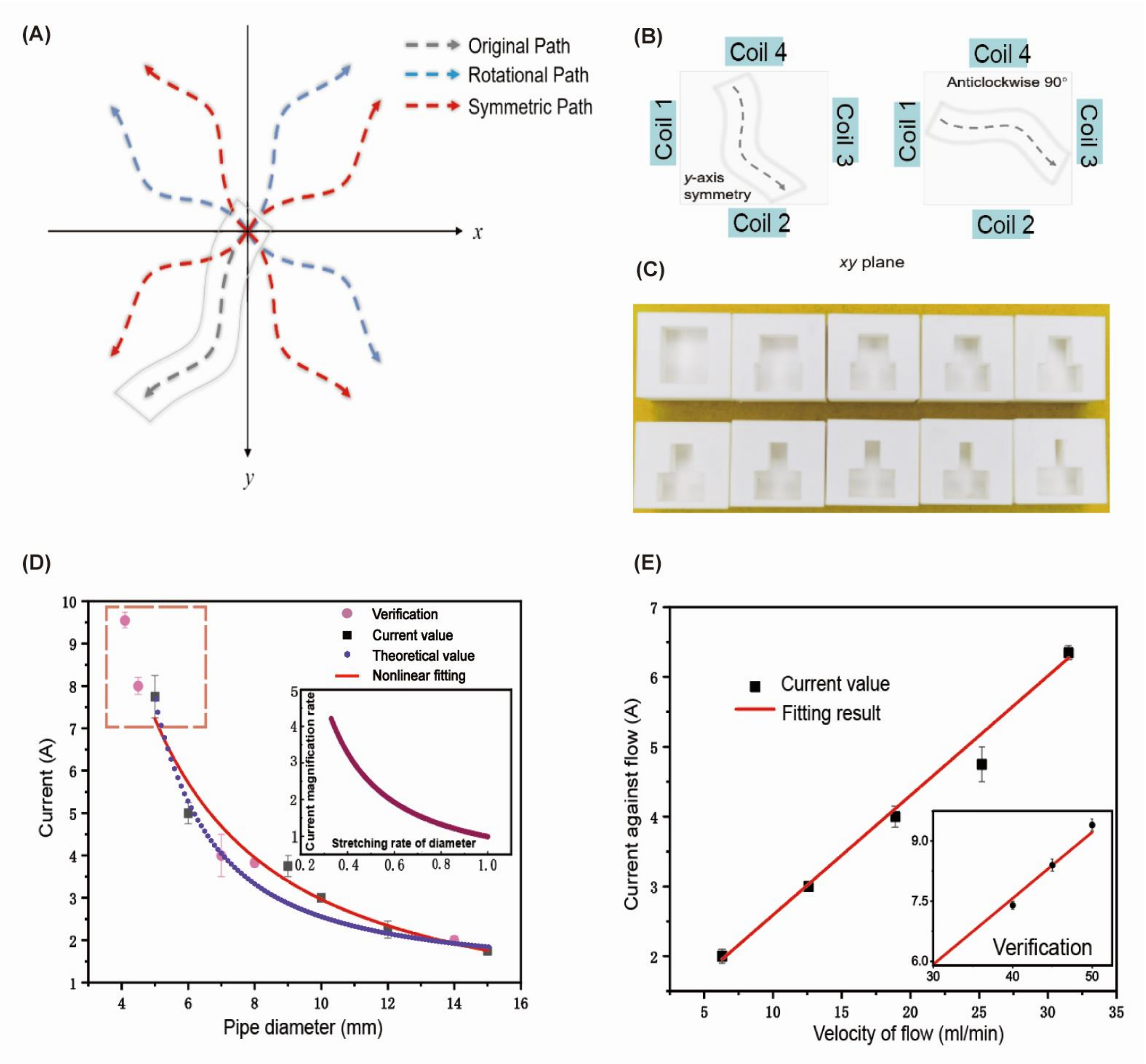

3.9 Expand the prediction to a more general environment

Supplementary Figure 9

(A) Rotational and symmetric operation of the original track. **(B)** The relative position of the track with the four coils. **(C)** The tracks for analyzing the current correction coefficient. **(D)** The current values needed to enter different diameters. **(E)** Current values needed to overcome the blood flow.

We perform rotating and symmetric operations on the original track and broaden the coordinate-current value through coordinates transformation. With the expanded coordinate-current data, we could broaden the training database to make the network capable of predicting over more complex paths. Supplementary Figure 7A shows all the rotating and symmetric operations. Supplementary Figure 7B shows the relative position of the transformed track with the 4 coils, the entire transforming operation could nearly cover the whole platform. To make sure the current values associated with the position coordinates can be completely planted after rotation, we restrict the rotating angle to be 90° and its multiples.

Supplementary Figure 9C shows a series of small tracks to simulate the process of pipe diameter changing from large to small. Supplementary Figure 9D shows the current value needed to enter different vessel diameters. The black dots denote the experimental results, and the pink dots denote the verification. The purple curve is the theoretical value of the drag coefficient of variation multiplied by the reference current, so it denots the theoretically required current value. The trend is consistent with the measurements, with slightly smaller values. This result is reasonable because the actual experiment requires an applied magnetic force greater than the resistance to make the robot move, so the test value may be slightly larger. The verification results show that the experimental results are in good agreement with the fitted curve. Finally, supplementary figure 9E shows the current values required to keep the robot and the track relatively stationary at different flow rates. The embedded small graph verifies the universality of the fitting results.
